# Copy number variation associates with mortality in long‐lived individuals: a genome‐wide assessment

**DOI:** 10.1111/acel.12407

**Published:** 2015-10-08

**Authors:** Marianne Nygaard, Birgit Debrabant, Qihua Tan, Joris Deelen, Karen Andersen‐Ranberg, Anton J.M. de Craen, Marian Beekman, Bernard Jeune, Pieternella E. Slagboom, Kaare Christensen, Lene Christiansen

**Affiliations:** ^1^ The Danish Aging Research CenterEpidemiology, Biostatistics and BiodemographyDepartment of Public HealthUniversity of Southern DenmarkJ.B. Winsloews Vej 9B, 5000, Odense CDenmark; ^2^ Department of Clinical GeneticsOdense University HospitalSdr. Boulevard 29, 5000, Odense CDenmark; ^3^ Department of Molecular EpidemiologyLeiden University Medical CenterP.O. Box 9600, 2300 RC LeidenThe Netherlands; ^4^ Department of Gerontology and GeriatricsLeiden University Medical CenterP.O. Box 9600, 2300 RC LeidenThe Netherlands; ^5^ Department of Clinical Biochemistry and PharmacologyOdense University HospitalSdr. Boulevard 29, 5000 Odense CDenmark

**Keywords:** aging, copy number variation, deletions, genetics, long‐lived individuals, mortality

## Abstract

Copy number variants (CNVs) represent a significant source of genetic variation in the human genome and have been implicated in numerous diseases and complex traits. To date, only a few studies have investigated the role of CNVs in human lifespan. To investigate the impact of CNVs on prospective mortality at the extreme end of life, where the genetic component of lifespan appears most profound, we analyzed genomewide CNV data in 603 Danish nonagenarians and centenarians (mean age 96.9 years, range 90.0–102.5 years). Replication was performed in 500 long‐lived individuals from the Leiden Longevity Study (mean age 93.2 years, range 88.9–103.4 years). First, we assessed the association between the CNV burden of each individual (the number of CNVs, the average CNV length, and the total CNV length) and mortality and found a significant increase in mortality per 10 kb increase in the average CNV length, both for all CNVs (hazard ratio (HR) = 1.024, *P* = 0.002) and for duplications (HR = 1.011, *P* = 0.005), as well as per 100 kb increase in the total length of deletions (HR = 1.009, *P* = 0.0005). Next, we assessed the relation between specific deletions and duplications and mortality. Although no genome–wide significant associations were discovered, we identified six deletions and one duplication that showed consistent association with mortality in both or either of the sexes across both study populations. These results indicate that the genome–wide CNV burden, specifically the average CNV length and the total CNV length, associates with higher mortality in long‐lived individuals.

## Introduction

Human lifespan is a complex trait with a moderate heritability of around 25% (Herskind *et al*., 1996). So far, only a limited number of loci have been consistently associated with length of life (Murabito *et al*., 2012; Deelen *et al*., 2014), and hence much of the heritability is still unaccounted for. This missing heritability might in part be explained by copy number variants (CNVs) (Manolio *et al*., 2009).

Copy number variants are defined as DNA segments larger than 1 kb in size that vary in copy number between individuals due to insertion, deletion, or duplication (Feuk *et al*., 2006a). The mechanisms by which CNVs can affect gene expression and phenotypic traits include alteration of gene dosage and disruption of coding sequences or regulatory elements (Feuk *et al*., 2006b). CNVs have been implicated in numerous diseases and complex traits, like schizophrenia (International Schizophrenia C, 2008), autism (Marshall *et al*., 2008), and obesity (Wheeler *et al*., 2013), but only a few studies have addressed the role of CNVs in survival and longevity.

The first study of CNVs related to human lifespan was performed by Kuningas *et al*. (2011), who prospectively studied 11 442 individuals with an intake age of 34.0–75.3 years and found that carriers of a higher burden of common deletions larger than 500 kb had a marginally increased risk of mortality [hazard ratio (HR) = 1.04] compared to noncarriers. They also identified two common CNV regions (CNVRs) on chromosome 11p15.5 and 14q21.3 that associated with higher mortality at old age. Next, Glessner and colleagues compared CNV frequencies between 7313 pediatric (aged 0–18 years) and 2701 geriatric individuals (aged > 67 years, mean age 76 years) with replication in 2079 children (mean age 8.6 years) and 4692 elderly adults (mean age 60 years). In total, they identified three deletions and four duplications that were significantly enriched in the pediatric individuals and that primarily encompassed genes involved in RNA splicing, suggesting an impact of this biological mechanism on lifespan (Glessner *et al*., 2013). Recently, an analysis of the association between 20 common CNVs not typically covered by commercial arrays and longevity in 222 senior American Caucasians with replication in 1283 community‐dwelling senior European Caucasians resulted in the identification of a deletion in the neurexin superfamily member *CNTNAP4* on chromosome 16q23.1 that was inversely associated with survival to 80 years in women (Iakoubov *et al*., 2013). Further investigation of CNVs in other gene members of the neurexin superfamily by the same group additionally led to the detection of a CNV in the *CNTNAP2* gene on chromosome 7q35‐36.1 that negatively associated with healthy aging in octogenarian men (Iakoubov *et al*., 2015).

None of the studies performed so far have focused on the impact of CNVs on mortality at the extreme end of life. Here, we explored this by analyzing the association between the CNV burden of each individual and prospective mortality, as well as between specific deletions and duplications and prospective mortality in long‐lived individuals of Danish descent (DKLS, mean age 96.9 years, range 90.0–102.5 years). The long‐lived individuals are all older than 90 years, and they thus represent the age group where the influence of genetic factors on survival is likely to be most profound (Hjelmborg *et al*., 2006). Suggestive findings were replicated in a second population of long‐lived individuals from the Leiden Longevity Study (LLS, mean age 93.2 years, range 88.9–103.4 years), and, in addition, a joint analysis of these findings was performed.

## Results

The characteristics of the discovery study (DKLS) and the replication study (LLS) populations are summarized in Table 1.

**Table 1:** Study population characteristics

	Discovery study (DKLS)	Replication study (LLS)
*N*, total	603	500
*N*, women (%)	453 (75.1%)	302 (60.4%)
Mean age at baseline (range)	96.9 (90.0–102.5)	93.2 (88.9–103.4)
Mean follow‐up time (range)	2.9 (0.01–12.9)	4.1 (0.02–11.5)
*N*, deaths (%)	521 (86.4%)	457 (91.4%)

Applying a Bonferroni‐corrected significance level of 0.006, the analysis of the CNV burden of each individual revealed a significant association between a longer average CNV length and increased mortality. In the joint analysis including both the DKLS and the LLS, this was evident for the combined group of CNVs where we detected an increase in HR of 2.4% per 10 kb increase in the average CNV length (HR = 1.024, *P* = 0.002) as well as for duplications only (HR = 1.011, *P* = 0.005) (see Table 2). In addition, we observed a significant increase in mortality as a result of a greater part of the genome being occupied by deletions with the HR increasing 0.9% per 100 kb increase in the total length of deletions (HR = 1.009, *P* = 0.0005).

**Table 2:** Association between the copy number variant (CNV) burden of each individual and mortality

		All CNVs		Deletions		Duplications	
		HR (95% CI)	*P*	HR (95% CI)	*P*	HR (95% CI)	*P*
Number of CNVs^a^	Discovery study (DKLS)	0.991 (0.919–1.068)	0.811	1.049 (0.957–1.150)	0.307	0.917 (0.814–1.034)	0.158
	Replication study (LLS)	1.001 (0.928–1.080)	0.997	1.163 (0.959–1.409)	0.124	0.985 (0.912–1.063)	0.697
	Joint analysis	0.996 (0.944–1.050)	0.879	1.069 (0.984–1.162)	0.113	0.965 (0.905–1.029)	0.275
Average CNV length^b^	Discovery study (DKLS)	1.025 (1.009–1.041)	0.002	1.015 (1.002–1.027)	0.023	1.011 (1.003–1.020)	0.008
	Replication study (LLS)	1.012 (0.963–1.062)	0.644	1.007 (0.980–1.035)	0.598	1.009 (0.977–1.042)	0.597
	Joint analysis	1.024 (1.009–1.039)	**0.002**	1.014 (1.003–1.025)	0.013	1.011 (1.003–1.019)	**0.005**
Total CNV length^c^	Discovery study (DKLS)	1.006 (0.998–1.015)	0.146	1.011 (1.000–1.023)	0.050	1.001 (0.989–1.014)	0.814
	Replication study (LLS)	1.004 (0.993–1.015)	0.523	1.009 (1.004–1.014)	0.001	0.999 (0.985–1.014)	0.911
	Joint analysis	1.005 (0.999–1.012)	0.107	1.009 (1.004–1.015)	**0.0005**	1.000 (0.991–1.009)	0.973

HR, hazard ratio; 95% CI, 95% confidence interval; *P*,* P*‐value obtained from a Cox proportional hazard regression adjusted for study relevant covariates or from the joint analysis.

The *P*‐value is not adjusted for multiple testing. Joint analysis *P*‐values ≤ 0.006 are shown in bold.

a Results are per additional 10 CNVs.

b Results are per additional 10 kb.

c Results are per additional 100 kb.

Altogether, a total of 272 unique deletions and 131 unique duplications were identified in the DKLS. This distribution of deletions and duplications is unlikely to reflect the actual distribution of CNVs in the human genome and rather represents a detection bias associated with the sensitivity of the CNV detection algorithm used (Zheng *et al*., 2012). In the association analysis, none of the CNVs reached statistical significance after correcting for multiple testing. However, when using a nominal significance level of *P* ≤ 0.05, 20 CNVs were found to associate with mortality (see Table 3), and they were thus selected for replication in the LLS. In the joint analysis including both study populations, the association with mortality was consistent for three deletions located on chromosome 6q14.1 (HR 1.35, 95% CI 1.04–1.74, *P* = 0.023), 10p13 (HR 1.67, 95% CI 1.05–2.66, *P* = 0.031), and 13q13.2 (HR 1.43, 95% CI 1.07–1.91, *P* = 0.015) and one duplication located on chromosome 9q21.12 (HR 1.55, 95% CI 1.07–2.25, *P* = 0.021). All of these CNVs were found to contribute to a higher mortality in carriers compared to noncarriers. Furthermore, sex‐specific analyses (see Table S1 and S2, Supporting Information) revealed three additional deletions on chromosome 1p22.3, 5q31.3, and 8p22 that resulted in higher mortality among carriers in one of the sexes.

**Table 3:** Association between specific deletions and duplications and mortality

				Discovery study (DKLS)			Replication study (LLS)			Joint analysis	
Copy number variant^a^	Locus	Type	Size (kb)	Freq. (%)	HR (95% CI)	*P*	Freq. (%)	HR (95% CI)	*P*	HR (95% CI)	*P*
Chr2:18327703‐18351537	2p24.2	DUP	23.8	1.3	3.48 (1.62–7.45)	0.001	1.2	0.85 (0.52–1.38)	0.512	1.28 (0.85–1.94)	0.239
Chr9:9516348‐9526811	9p23	DUP	10.5	1.0	3.30 (1.45–7.53)	0.005	NA	NA	NA	NA	NA
Chr13:32896846‐32922331	13q13.1	DEL	25.5	1.3	2.80 (1.30–6.03)	0.009	NA	NA	NA	NA	NA
Chr1:80982632‐81056073	1p31.1	DUP	73.4	1.0	2.97 (1.29–6.80)	0.010	NA	NA	NA	NA	NA
Chr6:55828727‐55846527	6p12.1	DEL	17.8	3.3	1.83 (1.14–2.95)	0.012	NA	NA	NA	NA	NA
Chr10:13055619‐13058458	10p13	DEL	2.8	2.0	2.08 (1.17–3.72)	0.013	1.2	1.15 (0.53–2.47)	0.729	1.67 (1.05–2.66)	**0.031**
Chr6:29855945‐29909559	6p22.1	DEL	53.6	11.8	1.38 (1.06–1.79)	0.015	5.0	0.87 (0.55–1.39)	0.565	1.24 (0.99–1.56)	0.057
Chr9:73902148‐73909871	9q21.12	DUP	7.7	1.8	2.05 (1.12–3.75)	0.020	4.0	1.31 (0.81–2.10)	0.272	1.55 (1.07–2.25)	**0.021**
Chr8:8583109‐8589783	8p23.1	DEL	6.7	1.5	2.18 (1.12–4.26)	0.022	1.0	0.93 (0.23–3.75)	0.918	1.87 (1.02–3.43)	0.042
Chr8:30990110‐31000860	8p12	DEL	10.8	1.0	2.60 (1.14–5.94)	0.023	NA	NA	NA	NA	NA
Chr13:34115595‐34143545	13q13.2	DEL	28.0	7.8	1.45 (1.05–2.00)	0.023	2.6	1.32 (0.64–2.70)	0.453	1.43 (1.07–1.91)	**0.015**
Chr15:102028468‐102305129	15q26.3	DUP	276.7	1.0	2.52 (1.12–5.69)	0.026	NA	NA	NA	NA	NA
Chr20:36051525‐42681088	20q11.23, 20q12, 20q13.11, 20q13.12	DEL	6629.6	1.8	0.45 (0.22–0.91)	0.026	1.0	0.89 (0.47–1.70)	0.729	0.65 (0.40–1.05)	0.079
Chr7:118006251‐118070496	7q31.31	DEL	64.2	1.0	2.69 (1.11–6.56)	0.029	NA	NA	NA	NA	NA
Chr6:31275246‐31285292	6p21.33	DEL	10.0	2.8	1.76 (1.05–2.96)	0.032	2.4	1.02 (0.67–1.56)	0.923	1.27 (0.92–1.74)	0.150
Chr13:88960710‐89033066	13q31.2	DEL	72.4	1.0	2.38 (1.06–5.37)	0.036	NA	NA	NA	NA	NA
Chr22:18844632‐19016663	22q11.21	DUP	172.0	1.3	2.11 (1.04–4.28)	0.039	NA	NA	NA	NA	NA
Chr1:161490896‐161617516	1q23.3	DUP	126.6	1.8	0.48 (0.23–0.98)	0.043	1.4	2.60 (1.35–5.04)	0.004	1.17 (0.72–1.90)	0.520
Chr6:77439969‐77448731	6q14.1	DEL	8.8	6.5	1.42 (1.01–2.00)	0.046	4.4	1.27 (0.88–1.84)	0.194	1.35 (1.04–1.74)	**0.023**
Chr13:82081150‐82087510	13q31.1	DEL	6.4	1.0	2.28 (1.01–5.14)	0.048	NA	NA	NA	NA	NA

a Copy number variant (CNV), The discovery study CNV position with information about chromosome and start and stop base pair positions based on the GRCh37/hg19 genome build; Freq., frequency; HR, hazard ratio; 95% CI, 95% confidence interval; *P*,* P*‐value obtained from a Cox proportional hazard regression adjusted for study relevant covariates or from the joint analysis.

The *P*‐value is not adjusted for multiple testing. Joint analysis *P*‐values ≤ 0.05 for variants showing the same direction of effect in the discovery and replication studies are shown in bold. NA, not applicable due to a frequency lower than 1% in the replication study.

The seven nominally significant CNVs have all, at least partially, been reported by the Database of Genomic Variants (DGV, http://dgv.tcag.ca/dgv/app/home; Macdonald *et al*., 2014), supporting that they represent true findings. They encompass or are positioned nearby a number of genes: *COL24A1*,* PCDHA1*‐*PCDHA10*,* IMPG1*,* IRAK1BP1*,* PSD3*,* TRPM3*,* TMEM2*,* CCDC3,* and *STARD13* (see Table S3). These genes are significantly enriched for several Gene Ontology (GO) terms related to cell adhesion (hemophilic cell adhesion via plasma membrane adhesion molecules, cell–cell adhesion via plasma membrane adhesion molecules, cell–cell adhesion, cell adhesion, and biological adhesion) as well as for the GO term nervous system development. However, when limiting the contribution of the 10 *PCDHA* genes by including only one of them in the enrichment analysis, no GO terms are significantly over‐ or underrepresented. In addition to genes, the 5q31.3, 8p22, and 13q13.2 deletions all include CpG islands and regions with indications of active regulatory elements. The 5q31.3 deletion also contains a number of eQTLs consisting of single nucleotide polymorphisms (SNPs) that show association with the expression of the *WDR55* and *ARL4A* genes, primarily in brain tissue.

## Discussion

In this study, we explored the impact of copy number variation on mortality at the extreme end of life by performing a genome‐wide investigation of the association between CNVs and prospective mortality in nonagenarians and centenarians.

As our main result, we found that an increase in the average CNV length significantly associated with a higher mortality, as did an increase in the total part of the genome occupied by deletions (see Table 2). These findings are consistent with the results of a previous study in which the burden of large deletions was found to be associated with higher mortality (Kuningas *et al*., 2011), suggesting that longer CNVs, especially deletions, are more disadvantageous. The identified association between a higher CNV burden and increased mortality is generally in line with the proposed role of genome instability, that is, a decrease in genome maintenance and hence an accumulation of genomic changes, in lifespan (Vijg & Suh, 2013) and suggests that even among the very old, the load of genomic alterations is linked to differences in mortality.

Among the specific deletions and duplications, three deletions on chromosome 6q14.1, 10p13, and 13q13.2 and one duplication on chromosome 9q21.12 were consistently associated with higher mortality across the DKLS and LLS populations (see Table 3), as were a deletion on chromosome 5q31.3 in women (see Table S1) and two deletions on chromosome 1p22.3 and 8p22 in men (see Table S2). These seven nominally significant CNVs are surrounded by numerous genes (see Table S3), of which two, *TRPM3* and *STARD13*, have previously been implicated in the regulation of human lifespan (Lunetta *et al*., 2007; Yashin *et al*., 2010). The *STARD13* gene has moreover been associated (*P* ≤ 1 × 10^−5^) with plasma levels of amyloid beta peptides that, among other things, play a role in Alzheimer's disease and hypertension (Chouraki *et al*., 2014). In addition, also the *CCDC3* and *IRAK1BP1* genes could be speculated to play a role in human lifespan, as they have been reported to inhibit inflammation (Conner *et al*., 2008, 2010; Azad *et al*., 2014), and as revealed by the GO enrichment analysis, the majority of the other genes are involved in cell adhesion, which has previously been linked to longevity and age‐related diseases (Wolfson *et al*., 2009; Tian *et al*., 2013).

In addition to their more direct effect on genes, for example, alteration of gene dosage and gene disruption, CNVs may also affect regulatory regions or other functional regions that influence gene expression, and CNVs have indeed been reported to exert their effect over distances of more than 6 Mb (Stranger *et al*., 2007). Only a few of the seven CNVs found to potentially associate with mortality in long‐lived individuals in this study contain known regulatory elements. The CNVs harboring these elements additionally overlap or include genes, and especially the 5q31.3 region, which includes the genes *PCDHA1‐PCDHA10*, contains a substantial number of CpG islands. The eQTL analysis revealed that this region also affects the expression of the *WRD55* and *ARL4A* genes, primarily in brain tissue. Epigenetic changes in *ARL4A* have previously been found to associate with maternal longevity in an epigenome‐wide association study of age and age‐related phenotypes (Bell *et al*., 2012), indicating a possible link between the 5q31.3 deletion and longevity.

A number of genes known to be of interest for longevity are included in the CNVs associated with mortality in the DKLS. For instance, a deletion in *WRN* is found to be associated with higher mortality. The *WRN* gene encodes a RecQ DNA helicase involved in DNA replication and repair, transcription, and telomere maintenance (Rossi *et al*., 2010). Deleterious mutations in this gene give rise to the premature aging syndrome Werner, and genetic variation in *WRN* has previously been associated with longevity (Soerensen *et al*., 2012). Also worth highlighting are the genes *SGK2*, which is part of the insulin/insulin‐like growth factor 1 (IGF‐1) pathway and has been implicated in human longevity (Deelen *et al*., 2013), and *BRCA2* that is involved in DNA double‐strand break repair (Jensen, 2013) and has been found to play a role in the lifespan of mice (Donoho *et al*., 2003). These CNVs were, however, not attempted replicated, due to a very low frequency (below 1%) in the LLS sample.

Two studies have recently investigated the potential impact of CNVs on survival and longevity in a genome‐wide manner (Kuningas *et al*., 2011; Glessner *et al*., 2013). We did not find any overlap in results between our study and the study by Glessner and co‐workers. However, as this study is a case–control study comparing elderly individuals, primarily in their sixties and seventies, with young individuals under the age of 19, and as the significantly associated CNVs identified were enriched only in the pediatric individuals, a lack of overlap could be expected. In contrast, we saw a minor overlap, although only in sex‐specific analyses, of two CNVs with the study by Kuningas and colleagues, which is the only other longitudinal study on CNVs and mortality published so far. The overlapping CNVs are a duplication on chromosome 14q32.33 and a deletion on chromosome 14q21.2. Due to the use of a different genome build, the 14q21.2 region is reported as 14q21.3 by Kuningas and co‐workers. In both instances, the direction of effect is the same, that is, for the 14q21.2 deletion HR > 1 and for the 14q32.33 duplication HR < 1, which further supports a role for these genetic variations in mortality during old age.

There are a number of possible explanations for the modest agreement between our study and the study by Kuningas and co‐workers. For one, the intake age range of the populations included in the two studies is different (88.9–103.4 years vs. 34.0–75.3 years, respectively). This means that while Kuningas and colleagues focus on mortality from middle age to old age, our study investigates mortality at very advanced ages. Thus, we explore the possible significance of CNVs in individuals belonging to the age group where the genetic component of longevity has been suggested to be most profound and hence more profound that at younger ages (Hjelmborg *et al*., 2006). In agreement with this, it has previously been shown that the association between the *APOE* ε4 risk allele and mortality increases with higher ages, even after the age of 100 (Jacobsen *et al*., 2010). Additionally, the advanced age of our study population means that a larger part of the individuals included in our study died during follow‐up, which results in a relatively high number of events and limits the potential bias associated with censoring. Other parameters affecting the ability to replicate initial findings could be related to differences in sample size and genetic background, the use of different platforms and CNV detection algorithms as well as potential technical and random errors. Compared to the study by Kuningas and colleagues, our sample size is smaller, and our power to detect CNVs with lower effect sizes is therefore reduced. However, despite our smaller sample size, we still detect a higher number of CNVRs (403 vs. 312). Although these numbers are not entirely comparable due to the use of different methods for defining the CNVRs, this difference is likely to partly reflect their use of an array containing a lower number of SNPs, and hence with a lower resolution, than ours.

In conclusion, we found that the genomewide CNV burden, specifically the average CNV length and the total CNV length, associates with higher mortality in long‐lived individuals. Our results indicate that CNVs might be important contributors to the genetic component of human longevity and prompt further investigation.

## Experimental procedures

### Study populations

#### Discovery study

The discovery study population, also referred to as the Danish Longevity Study (DKLS), consisted of participants drawn from the Longitudinal Study of Danish Centenarians (LSDC), the 1905 Birth Cohort Study, the 1911–12 Birth Cohort Study, the Study of Danish Old Sibs (DOS), and the Longitudinal Study of Ageing Danish Twins (LSADT). Briefly, the LSDC and the Danish 1905 Birth Cohort Study are prospective follow‐up studies initiated when participants were 100 and 92 years of age, respectively (Andersen‐Ranberg *et al*., 2001; Nybo *et al*., 2001), the Danish 1911–12 Birth Cohort Study (Robine *et al*., 2010; Vestergaard *et al*. 2015) consists of individuals attaining the age of 100 in the period from May 5th, 2011 to July 5th, 2012, DOS was initiated in 2004 and includes families in which at least two siblings were ≥ 90 years of age at intake, and LSADT was initiated in 1995 and includes Danish twins ≥ 70 years of age (Skytthe *et al*., 2002).

From DOS and LSADT, one individual from each sib‐ship or twin pair was selected among participants that had reached an age of at least 91 years for DOS and 90 years for LSADT. From the 1905 Birth Cohort Study, participants were selected among individuals reaching an age of minimum 96 years. To avoid immortal follow‐up time, individuals from DOS, LSADT, and the 1905 Birth Cohort Study were followed from age 91, 90, and 96 onwards.

#### Replication study

The replication study population consisted of nonagenarian individuals from the LLS. In the LLS, long‐lived siblings of European descent were recruited together with their offspring and the spouses of the offspring. Families were included if at least two long‐lived siblings were alive and fulfilled the age criterion of 89 years or older for men and 91 years or older for women, representing < 0.5% of the Dutch population in 2001 (Schoenmaker *et al*., 2006). In total, 944 long‐lived proband siblings (mean age 94 years, range 89–104 years), 1671 offspring (mean age 61 years, range 39–81 years), and 744 offspring partners (mean age 60 years, range 36–79 years) were included. Here, only long‐lived probands (*N* = 500), who were previously genotyped using Illumina HumanOmniExpress BeadChips (Illumina Inc., San Diego, CA, USA), were included.

Vital status was followed until death, January 1st, 2014 (in the DKLS), or February 1st, 2014 (in the LLS), whichever came first. Information on survival status was retrieved from the Danish (Pedersen *et al*., 2006) and Dutch Central Population Registers.

In the DKLS, permissions to collect blood samples and the use of register‐based data were granted by the Danish National Committee on Biomedical Research Ethics. The LLS was approved by the Medical Ethical Committee of the Leiden University Medical Center, and all participants gave written informed consent.

### Genotyping and quality control

In the DKLS, DNA was isolated from whole blood using standard methods (Miller *et al*., 1988) and genotyping was carried out according to the manufacturer's protocol using the Illumina HumanOmniExpress BeadChips (Illumina Inc.) (Deelen *et al*., 2014), which contains 730 525 SNP markers with a median spacing of 2.1 kb and a mean spacing of 4.0 kb.

Quality control (QC) was performed in Plink (http://pngu.mgh.harvard.edu/~purcell/plink/; Purcell *et al*., 2007) and samples were evaluated and excluded based on call rate (call rate < 98.5%), inconsistencies between genotypic and phenotypic sex, and family relationships (proportional IBD > 0.1875). Of the 673 originally included samples, 662 samples remained after the genotype‐based QC.

### CNV detection and QC

Copy number variants were detected on autosomes only using the PennCNV software (Wang *et al*., 2007). The hidden Markov model implemented in PennCNV incorporates multiple sources of information, including the total signal intensity [log R ratio (LRR)] and the allelic intensity ratio [B allele frequency (BAF)] of each SNP marker, the distance between the SNPs, and the population frequency of the B allele (PFB) of SNPs. LRR and BAF values were exported from GenomeStudio (Illumina Inc.) and the PFB file was compiled from the DKLS samples based on the BAF of each marker.

After CNV detection, samples with a LRR standard deviation > 0.30 were excluded, as were samples with more than 100 CNVs called and samples with a GC wave factor above 0.05 or below −0.05. Altogether, 59 samples were excluded, resulting in the inclusion of 603 DKLS samples in the final analysis.

Copy number variants were excluded if they were < 1 kb in size or contained < 3 consecutive SNPs indicating a deletion or a duplication.

### Generation of CNV datasets

Data on CNV burden, that is, the number of CNVs, the average CNV length, and the total CNV length, were retrieved for each individual using Plink (http://pngu.mgh.harvard.edu/~purcell/plink/; Purcell *et al*., 2007).

The data on specific deletions and duplications were constructed by merging CNVs of the same state [either deletion (state 0 or 1) or duplication (state 3 or 4)] if they overlapped by at least 50% of the length of the shorter CNV. The outermost boundaries of the merged regions were used to define the CNVs.

Copy number variants overlapping centromeres or telomeres (as defined by UCSC (http://genome.ucsc.edu/) using the Table Browser Software (Karolchik *et al*., 2004) and the GRCh37/hg19 genome build) were excluded, as were CNVs with a frequency < 1%.

### Replication of CNV associations

Copy number variants of the same state overlapping nominally significant findings from the discovery study were evaluated for association with mortality in the LLS individuals (*N* = 500), who were previously genotyped using the same SNP array as in the DKLS (Illumina HumanOmniExpress BeadChips; Illumina Inc.).

DNA from the LLS was isolated from white blood cells using conventional methods (Beekman *et al*., 2006). To ensure a high comparability between the DKLS and the LLS, genotyping and CNV detection in the LLS were carried out as described for the DKLS with only minor differences. In the genotype‐based QC, samples were excluded based on a call rate < 95% and no evaluation of family relationships was performed. For the CNV detection, a standard PFB file (OmniExpress_hg19.pfb.new), available on the PennCNV website (http://www.openbioinformatics.org/penncnv/), was used. In addition, a modified GC‐model file based on hg19 was included.

### Statistical analysis

All survival analyses were carried out using the statistical software Stata (Stata version 13.1; Stata Corporation, College Station, TX, USA) by applying a left‐truncated Cox proportional hazard regression model adjusted for sex, age at baseline, birth cohort (in the DKLS only), and familial relations (in the LLS only). The adjustment for birth cohort in the DKLS was included in order to account for any confounding due to differences in survival probability between the birth cohorts, which has been shown to be important in genetic studies (Nygaard *et al*., 2014). The adjustment was carried out by including a categorical variable based on 10‐year birth year intervals in the model.

The joint analysis was performed in Plink (http://pngu.mgh.harvard.edu/~purcell/plink/; Purcell *et al*., 2007) as a meta‐analysis using a fixed‐effect approach. The HRs and the corresponding standard errors were calculated within each study and combined to obtain a joint analysis HR and *P*‐value. The 95% confidence intervals of the joint analysis HRs were calculated using R (http://www.r-project.org/).

In the CNV burden analysis, the association between the number of CNVs, the average CNV length, and the total CNV length of each individual and mortality was analyzed for all CNVs as well as for deletions only and duplications only in the DKLS, in the LLS, and in a joint analysis including the two populations. Given that the total CNV length is more or less the product of the number of CNVs and the average CNV length, that ‘all CNVs’ is made up of deletions and duplications, and that the joint analysis includes the DKLS and the LLS, a Bonferroni‐corrected significance level of *P* ≤ 0.006 was applied. This corresponds to the correction for eight tests, that is, assessing the association between the number of CNVs and the average CNV length of each individual and mortality for deletions only and duplications only in the DKLS and in the LLS.

In the association analysis of specific deletions and duplications and mortality, a *P*‐value ≤ 1.2 × 10^−4^ (0.05/403) was used as the cutoff for statistical significance. CNVs associated with mortality with a *P*‐value lower or equal to 0.05 were considered suggestive associations and were attempted replicated as well as included in the joint analysis.

### Gene ontology enrichment analysis and expression quantitative trait loci (eQTL) analysis

The biological processes associated with the genes encompassed by or surrounding the CNVs found to suggestively associate with mortality in the DKLS and the LLS in both genders combined or in women or men only was assessed using the GO enrichment analysis tool offered by the GO Consortium (http://geneontology.org/page/go-enrichment-analysis).

The potential effects on gene expression of SNPs in the CNVs suggestively associated with mortality were examined using the Genotype‐Tissue Expression (GTEx) eQTL database (http://www.ncbi.nlm.nih.gov/gtex/GTEX2/gtex.cgi), which is based on expression data of brain (cerebellum, frontal cortex, temporal cortex, and pons), liver, and lymphoblastoid cell lines.

## Funding

This study was financially supported by the INTERREG 4 A programme Syddanmark‐Schleswig‐K.E.R.N (with EU funds from the European Regional Development Fund), the European Union's Seventh Framework Programme (FP7/2007‐2011) under grant agreement no. 259679, the VELUX Foundation, The National Program for Research Infrastructure 2007 (grant no. 09‐063256) from the Danish Agency for Science Technology and Innovation, the US National Institute of Health (P01AG08761), the Danish Agency for Science, Technology and Innovation/The Danish Council for Independent Research (grant no. 11‐107308), and The Danish Interdisciplinary Research Council. The 5‐COOP project (5‐Country Oldest Old Project) is funded by the CERA Foundation (Lyon) and the AXA Research Fund, Paris, and The Health Foundation (Helsefonden), Copenhagen, Denmark. The LLS was supported by a grant from the Innovation‐Oriented Research Program on Genomics (SenterNovem IGE05007), the Centre for Medical Systems Biology, the Netherlands Consortium for Healthy Ageing (grant 050‐060‐810), and the Biobank‐Based Integrative Omics Studies (BIOS) Consortium funded by BBMRI‐NL, a research infrastructure financed by the Dutch government (NWO 184.021.007), all in the framework of the Netherlands Genomics Initiative, Netherlands Organization for Scientific Research (NWO).

## Conflict of interest

The authors declare no conflict of interest.

## Author contributions

Marianne Nygaard contributed to generation of study concept, study design, acquisition of data, data analysis, interpretation of data, and drafting the manuscript; Birgit Debrabant, to acquisition of data, data analysis, and interpretation of data; Qihua Tan, to acquisition of data, data analysis, and interpretation of data; Joris Deelen, to acquisition of data, data analysis, and interpretation of data; Karen Andersen‐Ranberg, to acquisition of data; Anton J. M. de Craen, to acquisition of data; Marian Beekman, to acquisition of data; Bernard Jeune, to acquisition of data; P. Eline Slagboom, to acquisition of data; Kaare Christensen, to acquisition of data and interpretation of data; Lene Christiansen, to generation of study concept, study design, acquisition of data, and interpretation of data; and all authors have revised the manuscript and given their final approval.

## Supporting information


**Table S1** Association between specific deletions and duplications and mortality in women.
**Table S2** Association between specific deletions and duplications and mortality in men.
**Table S3** Genes included in or surrounding the CNVs nominally associated with mortality (*P* ≤ 0.05) in the DKLS.
