# Supplementary material for: Copy number variation associates with mortality in long‐lived individuals: a genome‐wide assessment
Source: Aging Cell. 2015 Oct 8;15(1):49–55. doi: 10.1111/acel.12407 (PMC4717275; doi:10.1111/acel.12407)
Supplement: Supplementary file 1 — Table S1 Association between specific deletions and duplications and mortality in women. Table S2 Association between specific deletions and duplications and mortality in men. Table S3 Genes included in or surrounding the CNVs nominally associated with mortality (P ≤ 0.05) in the DKLS. [file ACEL-15-049-s001.docx]

**Supporting Information**

Table S1. Association between specific deletions and duplications and mortality in women.

|  | | | | **Discovery Study (DKLS)** | | | **Replication Study (LLS)** | | | **Joint Analysis** | |
| --- | --- | --- | --- | --- | --- | --- | --- | --- | --- | --- | --- |
| **CNV^1^** | **Locus** | **Type** | **Size (kb)** | **Freq. (%)** | **HR (95% CI)** | **P** | **Freq. (%)** | **HR (95% CI)** | **P** | **HR (95% CI)** | **P** |
| Chr2:18327703-18351537 | 2p24.2 | DUP | 23.8 | 1.6 | 3.92 (1.72-8.96) | 0.001 | 1.0 | 0.88 (0.41-1.88) | 0.733 | 1.76 (1.00-3.08) | 0.049 |
| Chr5:140165568-140237548 | 5q31.3 | DEL | 72.0 | 13.5 | 1.48 (1.11-1.99) | 0.008 | 1.0 | 1.81 (0.91-3.61) | 0.091 | 1.53 (1.17-2.00) | **0.002** |
| Chr3:99003734-99007270 | 3q12.1 | DEL | 3.5 | 1.1 | 3.17 (1.29-7.79) | 0.012 | NA | NA | NA | NA | NA |
| Chr20:36051525-42681088 | 20q11.23, 20q12, 20q13.11, 20q13.12 | DEL | 6629.6 | 2.0 | 0.36 (0.16-0.81) | 0.014 | 1.0 | 3.39 (1.71-6.71) | 0.0004 | 1.32 (0.78-2.22) | 0.301 |
|  |  |  |  |  |  |  | 1.0 | 1.13 (0.41-3.11) | 0.812 | 0.56 (0.30-1.06) | 0.073 |
| Chr6:55828727-55846527 | 6p12.1 | DEL | 17.8 | 3.3 | 1.93 (1.13-3.31) | 0.016 | NA | NA | NA | NA | NA |
| Chr1:161490896-161617516 | 1q23.3 | DUP | 126.6 | 2.0 | 0.37 (0.16-0.84) | 0.017 | 2.0 | 2.59 (1.27-5.29) | 0.009 | 1.14 (0.66-1.94) | 0.641 |
| Chr6:77439969-77448731 | 6q14.1 | DEL | 8.8 | 7.3 | 1.58 (1.09-2.29) | 0.017 | 3.3 | 1.11 (0.66-1.88) | 0.695 | 1.41 (1.04-1.91) | **0.028** |
| Chr9:73902148-73909871 | 9q21.12 | DUP | 7.7 | 1.6 | 2.45 (1.15-5.22) | 0.020 | 3.6 | 1.07 (0.63-1.82) | 0.808 | 1.42 (0.91-2.21) | 0.125 |
| Chr7:118006251-118070496 | 7q31.31 | DEL | 64.2 | 1.3 | 2.84 (1.16-6.95) | 0.022 | NA | NA | NA | NA | NA |
| Chr10:13055619-13058458 | 10p13 | DEL | 2.8 | 2.4 | 2.00 (1.09-3.67) | 0.025 | 1.0 | 1.49 (0.71-3.15) | 0.296 | 1.78 (1.11-2.85) | **0.017** |
| Chr9:9516348-9526811 | 9p23 | DUP | 10.5 | 1.1 | 2.73 (1.11-6.75) | 0.029 | NA | NA | NA | NA | NA |
| Chr8:8583109-8589783 | 8p23.1 | DEL | 6.7 | 1.6 | 2.31 (1.08-4.95) | 0.030 | NA | NA | NA | NA | NA |
| Chr13:32896846-32922331 | 13q13.1 | DEL | 25.5 | 1.6 | 2.51 (1.09-5.77) | 0.030 | NA | NA | NA | NA | NA |
| Chr8:115305708-115532721 | 8q23.3 | DEL | 227.0 | 1.3 | 2.70 (1.09-6.64) | 0.031 | 1.3 | 0.86 (0.27-2.69) | 0.794 | 1.74 (0.86-3.52) | 0.126 |
| Chr4:64099349-64155302 | 4q13.1 | DEL | 56.0 | 11.7 | 1.41 (1.02-1.94) | 0.036 | 7.0 | 0.95 (0.64-1.41) | 0.798 | 1.21 (0.95-1.54) | 0.129 |
| Chr6:29855945-29909559 | 6p22.1 | DEL | 53.6 | 12.4 | 1.36 (1.01-1.82) | 0.043  3 | 4.3 | 0.78 (0.41-1.48) | 0.445 | 1.24 (0.95-1.62) | 0.120 |
| Chr14:106047905-106326623 | 14q32.33 | DUP | 278.7 | 2.2 | 0.44 (0.19-0.99) | 0.047 | 31.1 | 0.99 (0.77-1.27) | 0.946 | 0.93 (0.74-1.16) | 0.520 |
| Chr6:77315788-77327833 | 6q14.1 | DEL | 12.0 | 2.0 | 0.41 (0.17-0.99) | 0.047 | NA | NA | NA | NA | NA |
| Chr14:19964076-20420338 | 14q11.2 | DUP | 456.3 | 17.4 | 0.77 (0.59-1.00) | 0.049 | 9.3 | 1.05 (0.77-1.42) | 0.762 | 0.88 (0.72-1.08) | 0.229 |

^1^CNV: The discovery study CNV position with information about chromosome and start and stop base pair positions based on the GRCh37/hg19 genome build. Freq.: Frequency. HR: Hazard ratio. 95% CI: 95% confidence interval. P: P-value obtained from a Cox proportional hazard regression adjusted for study relevant covariates or from the joint analysis. The P-value is not adjusted for multiple testing. Joint analysis P-values ≤ 0.05 for variants showing the same direction of effect in the discovery and replication studies are shown in bold. NA: Not applicable in the replication study due to a frequency lower than 1%.

Table S2. Association between specific deletions and duplications and mortality in men.

|  | | | | **Discovery Study (DKLS)** | | | **Replication Study (LLS)** | | | **Joint Analysis** | |
| --- | --- | --- | --- | --- | --- | --- | --- | --- | --- | --- | --- |
| **CNV^1^** | **Locus** | **Type** | **Size (kb)** | **Freq. (%)** | **HR (95% CI)** | **P** | **Freq. (%)** | **HR (95% CI)** | **P** | **HR (95% CI)** | **P** |
| Chr5:142767740-142786701 | 5q31.3 | DUP | 19.0 | 2.0 | 20.6 (5.12-82.7) | 0.00002 | NA | NA | NA | NA | NA |
| Chr2:189446471-189631806 | 2q32.2 | DUP | 185.3 | 1.3 | 100.4 (8.70-1158.7) | 0.0002 | NA | NA | NA | NA | NA |
| Chr8:18789374-18873422 | 8p22 | DEL | 84.0 | 1.3 | 32.5 (4.54-232.0) | 0.001 | 2.5 | 1.53 (0.66-3.54) | 0.318 | 2.46 (1.14-5.35) | **0.022** |
| Chr13:88960710-89033066 | 13q31.2 | DEL | 72.4 | 2.0 | 8.40 (2.31-30.5) | 0.001 | NA | NA | NA | NA | NA |
| Chr4:163838176-163854178 | 4q32.2 | DEL | 16.0 | 1.3 | 13.3 (2.63-67.3) | 0.002 | NA | NA | NA | NA | NA |
| Chr1:86402185-86412177 | 1p22.3 | DEL | 10.0 | 2.0 | 5.77 (1.68-19.8) | 0.005 | 1.0 | 2.92 (1.22-7.01) | 0.016 | 3.68 (1.79-7.53) | **0.0004** |
| Chr21:23654246-23661835 | 21q21.1 | DEL | 7.6 | 2.0 | 6.02 (1.71-21.2) | 0.005 | 4.5 | 1.24 (0.75-2.05) | 0.393 | 1.55 (0.97-2.49) | 0.068 |
| Chr11:28098605-28160335 | 11p14.1 | DEL | 61.7 | 1.3 | 8.87 (1.89-41.6) | 0.006 | NA | NA | NA | NA | NA |
| Chr11:31649475-31729792 | 11p13 | DEL | 80.3 | 1.3 | 8.05 (1.74-37.2) | 0.008 | NA | NA | NA | NA | NA |
| Chr22:18844632-19016663 | 22q11.21 | DUP | 172.0 | 2.7 | 4.08 (1.41-11.8) | 0.010 | NA | NA | NA | NA | NA |
| Chr11:31595748-31664340 | 11p13 | DEL | 68.6 | 2.7 | 4.31 (1.41-13.2) | 0.011 | NA | NA | NA | NA | NA |
| Chr19:20621828-20728777 | 19p12 | DEL | 106.9 | 6.0 | 2.48 (1.21-5.07) | 0.013 | 7.1 | 1.06 (0.59-1.91) | 0.851 | 1.49 (0.94-2.34) | 0.090 |
| Chr5:7169851-7191074 | 5p15.31 | DEL | 21.2 | 2.0 | 4.59 (1.35-15.6) | 0.014 | NA | NA | NA | NA | NA |
| Chr13:69213315-69276739 | 13q21.33 | DEL | 63.4 | 12.0 | 0.46 (0.25-0.87) | 0.017 | 5.1 | 1.89 (1.05-3.40) | 0.035 | 0.98 (0.64-1.50) | 0.911 |
| Chr11:14508698-14516906 | 11p15.2 | DEL | 8.2 | 1.3 | 6.18 (1.37-27.9) | 0.018 | NA | NA | NA | NA | NA |
| Chr2:213156498-213191389 | 2q43 | DEL | 34.9 | 6.7 | 2.21 (1.14-4.30) | 0.020 | 4.5 | 0.60 (0.26-1.40) | 0.238 | 1.34 (0.79-2.26) | 0.275 |
| Chr14:45181337-45214574 | 14q21.2 | DEL | 33.2 | 2.0 | 3.94 (1.18-13.2) | 0.026 | NA | NA | NA | NA | NA |
| Chr19:55245738-55362859 | 19q13.42 | DUP | 117.1 | 2.0 | 3.78 (1.15-12.5) | 0.029 | 1.5 | 1.09 (0.39-3.06) | 0.873 | 1.86 (0.85-4.08) | 0.121 |
| Chr6:78965525-79029367 | 6q14.1 | DEL | 63.8 | 22.0 | 0.60 (0.38-0.96) | 0.031 | 10.1 | 0.92 (0.56-1.50) | 0.732 | 0.74 (0.52-1.03) | 0.077 |
| Chr4:10396709-10404512 | 4p16.1 | DEL | 7.8 | 8.7 | 0.47 (0.24-0.94) | 0.033 | 7.6 | 1.25 (0.83-1.87) | 0.287 | 0.96 (0.68-1.37) | 0.843 |
| Chr7:17551902-17566678 | 7p21.1 | DEL | 14.8 | 1.3 | 4.95 (1.12-22.0) | 0.035 | NA | NA | NA | NA | NA |
| Chr17:77364581-77394037 | 17q25.3 | DUP | 29.5 | 1.3 | 4.91 (1.07-22.5) | 0.041 | 1.5 | 0.83 (0.32-2.17) | 0.704 | 1.37 (0.61-3.10) | 0.445 |
| Chr6:31219869-31228972 | 6p21.33 | DEL | 9.1 | 1.3 | 4.58 (1.05-19.9) | 0.042 | 3.5 | 0.69 (0.26-1.79) | 0.442 | 1.22 (0.54-2.72) | 0.634 |
| Chr15:61687640-61700296 | 15q22.2 | DEL | 12.7 | 2.7 | 2.96 (1.04-8.42) | 0.042 | NA | NA | NA | NA | NA |
| Chr12:31259372-31409778 | 12p11.21 | DUP | 150.4 | 8.7 | 1.86 (1.02-3.39) | 0.044 | 9.6 | 1.03 (0.59-1.77) | 0.926 | 1.36 (0.90-2.06) | 0.149 |
| Chr1:80982632-81056073 | 1p31.1 | DUP | 73.4 | 1.3 | 4.49 (1.02-19.7) | 0.046 | NA | NA | NA | NA | NA |
| Chr3:102529319-102635852 | 3q12.3 | DUP | 106.5 | 1.3 | 4.49 (1.02-19.7) | 0.046 | 1.5 | 0.40 (0.16-0.98) | 0.045 | 0.77 (0.36-1.67) | 0.515 |
| Chr5:84774104-84874542 | 5q14.3 | DUP | 100.4 | 1.3 | 4.49 (1.02-19.7) | 0.046 | NA | NA | NA | NA | NA |
| Chr13:48818485-48951025 | 13q14.2 | DUP | 132.5 | 1.3 | 4.49 (1.02-19.7) | 0.046 | NA | NA | NA | NA | NA |
| Chr15:24357212-24472002 | 15q11.2 | DEL | 114.8 | 4.0 | 0.36 (0.13-0.99) | 0.048 | NA | NA | NA | NA | NA |
| Chr5:97933888-97939616 | 5q15 | DEL | 5.7 | 1.3 | 4.34 (1.00-18.8) | 0.050 | NA | NA | NA | NA | NA |

^1^CNV: The discovery study CNV position with information about chromosome and start and stop base pair positions based on the GRCh37/hg19 genome build. Freq.: Frequency. HR: Hazard ratio. 95% CI: 95% confidence interval. P: P-value obtained from a Cox proportional hazard regression adjusted for study relevant covariates or from the joint analysis. The P-value is not adjusted for multiple testing. Joint analysis P-values ≤ 0.05 for variants showing the same direction of effect in the discovery and replication studies are shown in bold. NA: Not applicable in the replication study due to a frequency lower than 1%.

Table S3. Genes included in or surrounding the CNVs nominally associated with mortality (P ≤ 0.05) in the DKLS.

| **CNV^1^** | **Locus** | **Type** | **Size (kb)** | **Included Genes^2^** | **Surrounding Genes^3^** |
| --- | --- | --- | --- | --- | --- |
| Chr1:80982632-81056073 | 1p31.1 | DUP | 73.4 | *LOC101927412* |  |
| Chr1:86402185-86412177 | 1p22.3 | DEL | 10.0 | *COL24A1* |  |
| Chr1:161490896-161617516 | 1q23.3 | DUP | 126.6 | *HSPA6, FCGR3A, FCGR2C, FCGR3B* |  |
| Chr2:18327703-18351537 | 2p24.2 | DUP | 23.8 |  | *KCNS3, NT5C1B-RDH14* |
| Chr2:189446471-189631806 | 2q32.2 | DUP | 185.3 | *GULP1, DIRC1* |  |
| Chr2:213156498-213191389 | 2q43 | DEL | 34.9 | *ERBB4* |  |
| Chr3:99003734-99007270 | 3q12.1 | DEL | 3.5 |  | *DCBLD2, MIR548G* |
| Chr3:102529319-102635852 | 3q12.3 | DUP | 106.5 |  | *NFKBIZ, MIR548A3* |
| Chr4:10396709-10404512 | 4p16.1 | DEL | 7.8 |  | *WDR1, ZNF518B* |
| Chr4:64099349-64155302 | 4q13.1 | DEL | 56.0 |  | *LPHN3-AS1, TECRL* |
| Chr4:163838176-163854178 | 4q32.2 | DEL | 16.0 |  | *FSTL5, NAF1* |
| Chr5:7169851-7191074 | 5p15.31 | DEL | 21.2 |  | *PAPD7, LOC442132* |
| Chr5:84774104-84874542 | 5q14.3 | DUP | 100.4 |  | *EDIL3, NBPF22P* |
| Chr5:97933888-97939616 | 5q15 | DEL | 5.7 |  | *LINC01340, RGMB* |
| Chr5:140165568-140237548 | 5q31.3 | DEL | 72.0 | *SEE BELOW** |  |
| Chr5:142767740-142786701 | 5q31.3 | DUP | 19.0 | *NR3C1* |  |
| Chr6:29855945-29909559 | 6p22.1 | DEL | 53.6 | *HLA-H, HCG4B* |  |
| Chr6:31219869-31228972 | 6p21.33 | DEL | 9.1 |  | *HCG27, HLA-C* |
| Chr6:31275246-31285292 | 6p21.33 | DEL | 10.0 |  | *HLA-C, HLA-B* |
| Chr6:55828727-55846527 | 6p12.1 | DEL | 17.8 |  | *BMP5, COL21A1* |
| Chr6:77315788-77327833 | 6q14.1 | DEL | 12.0 |  | *IMPG1, IRAK1BP1* |
| Chr6:77439969-77448731 | 6q14.1 | DEL | 8.8 |  | *IMPG1, IRAK1BP1* |
| Chr6:78965525-79029367 | 6q14.1 | DEL | 63.8 |  | *IMPG1, IRAK1BP1* |
| Chr7:17551902-17566678 | 7p21.1 | DEL | 14.8 | *LOC101927630* |  |
| Chr7:118006251-118070496 | 7q31.31 | DEL | 64.2 |  | *ANKRD7, KCND2* |
| Chr8:8583109-8589783 | 8p23.1 | DEL | 6.7 |  | *CLDN23, MFHAS1* |
| Chr8:18789374-18873422 | 8p22 | DEL | 84.0 | *PSD3* |  |
| Chr8:30990110-31000860 | 8p12 | DEL | 10.8 | *WRN* |  |
| Chr8:115305708-115532721 | 8q23.3 | DEL | 227.0 |  | *CSMD3, TRPS1* |
| Chr9:9516348-9526811 | 9p23 | DUP | 10.5 | *PTPRD* |  |
| Chr9:73902148-73909871 | 9q21.12 | DUP | 7.7 |  | *TRPM3, TMEM2* |
| Chr10:13055619-13058458 | 10p13 | DEL | 2.8 | *CCDC3* |  |
| Chr11:14508698-14516906 | 11p15.2 | DEL | 8.2 | *COPB1* |  |
| Chr11:28098605-28160335 | 11p14.1 | DEL | 61.7 | *KIF18A, METTL15* |  |
| Chr11:31595748-31664340 | 11p13 | DEL | 68.6 | *ELP4* |  |
| Chr11:31649475-31729792 | 11p13 | DEL | 80.3 | *ELP4* |  |
| Chr12:31259372-31409778 | 12p11.21 | DUP | 150.4 |  | *DDX11, FAM60A* |
| Chr13:32896846-32922331 | 13q13.1 | DEL | 25.5 | *BRCA2* |  |
| Chr13:34115595-34143545 | 13q13.2 | DEL | 28.0 | *STARD13* |  |
| Chr13:48818485-48951025 | 13q14.2 | DUP | 132.5 | *ITM2B, LINC00441, RB1* |  |
| Chr13:69213315-69276739 | 13q21.33 | DEL | 63.4 | *PCDH9, LINC00550* |  |
| Chr13:82081150-82087510 | 13q31.1 | DEL | 6.4 |  | *LINC00377, SLITRK1* |
| Chr13:88960710-89033066 | 13q31.2 | DEL | 72.4 |  | *LINC00397, LINC00433* |
| Chr14:19964076-20420338 | 14q11.2 | DUP | 456.3 | *LOC100508046, OR4N2, OR4K1* |  |
| Chr14:45181337-45214574 | 14q21.2 | DEL | 33.2 |  | *FSCB, C14ORF28* |
| Chr14:106047905-106326623 | 14q32.33 | DUP | 278.7 | *ELK2AP* |  |
| Chr15:24357212-24472002 | 15q11.2 | DEL | 114.8 |  | *NDN, PWRN1* |
| Chr15:61687640-61700296 | 15q22.2 | DEL | 12.7 |  | *RORA, VPS13C* |
| Chr15:102028468-102305129 | 15q26.3 | DUP | 276.7 | *PCSK6, TM2D3, TARSL2* |  |
| Chr17:77364581-77394037 | 17q25.3 | DUP | 29.5 | *RBFOX3* |  |
| Chr19:20621828-20728777 | 19p12 | DEL | 106.9 | *ZNF737* |  |
| Chr19:55245738-55362859 | 19q13.42 | DUP | 117.1 | *SEE BELOW*** |  |
| Chr20:36051525-42681088 | 20q11.23, 20q12, 20q13.11, 20q13.12 | DEL | 6629.6 | *SEE BELOW**** |  |
| Chr21:23654246-23661835 | 21q21.1 | DEL | 7.6 |  | *LINC00308, D21S2088E* |
| Chr22:18844632-19016663 | 22q11.21 | DUP | 172.0 | *DGCR6, PRODH, DGCR5, DGCR9* |  |

* *PCDHA1, PCDHA2, PCDHA3, PCDHA4, PCDHA5, PCDHA6, PCDHA7, PCDHA8, PCDHA9, PCDHA10*

** *KIR3DL3, KIR2DL3, LOC101928804, KIR2DL1, KIR2DL4, KIR3DL1, KIR2DS4, KIR3DL2*

*** *BLCAP, LINC00489, LOC100287792, CTNNBL1, VSTM2L, RPRD1B, TGM2, LOC149684, SNHG11, RALGAPB, BPI, LBP, ADIG, ACTR5, SLC32A1, PPP1R16B, DHX35, FAM83D, LINC01370, MAFB, TOP1, LPIN3, PLCG1-AS1, PLCG1, CHD6, ZHX3, EMILIN3, PTPRT, LOC101927159, SRSF6, L3MBTL1, MYBL2, TOX2, SGK2, IFT52, GTSF1L*

^1^CNV: The discovery study CNV position with information about chromosome and start and stop base pair positions based on the GRCh37/hg19 genome build. ^2^Included Genes: Genes encompassed or overlapped by the CNV. Only validated or reviewed RefSeq are shown. ^3^Surrounding Genes: The nearest up- and down-stream gene. Only validated or reviewed RefSeq genes are shown.
